# The influence of trial order on learning from reward vs. punishment in a probabilistic categorization task: experimental and computational analyses

**DOI:** 10.3389/fnbeh.2015.00153

**Published:** 2015-07-24

**Authors:** Ahmed A. Moustafa, Mark A. Gluck, Mohammad M. Herzallah, Catherine E. Myers

**Affiliations:** ^1^School of Social Sciences and Psychology and Marcs Institute for Brain and Behaviour, University of Western SydneySydney, NSW, Australia; ^2^Department of Veterans Affairs, New Jersey Health Care SystemEast Orange, NJ, USA; ^3^Center for Molecular and Behavioral Neuroscience, Rutgers UniversityNewark, NJ, USA; ^4^Al-Quds Cognitive Neuroscience Lab, Palestinian Neuroscience Initiative, Faculty of Medicine, Al-Quds UniversityJerusalem, Palestine; ^5^Department of Pharmacology, Physiology and Neuroscience, Rutgers-New Jersey Medical SchoolNewark, NJ, USA; ^6^Department of Psychology, Rutgers University-NewarkNewark, NJ, USA

**Keywords:** category learning, reward, punishment, Q-learning computational model, intermixed trials

## Abstract

Previous research has shown that trial ordering affects cognitive performance, but this has not been tested using category-learning tasks that differentiate learning from reward and punishment. Here, we tested two groups of healthy young adults using a probabilistic category learning task of reward and punishment in which there are two types of trials (reward, punishment) and three possible outcomes: (1) positive feedback for correct responses in reward trials; (2) negative feedback for incorrect responses in punishment trials; and (3) no feedback for incorrect answers in reward trials and correct answers in punishment trials. Hence, trials without feedback are ambiguous, and may represent either successful avoidance of punishment or failure to obtain reward. In Experiment 1, the first group of subjects received an intermixed task in which reward and punishment trials were presented in the same block, as a standard baseline task. In Experiment 2, a second group completed the separated task, in which reward and punishment trials were presented in separate blocks. Additionally, in order to understand the mechanisms underlying performance in the experimental conditions, we fit individual data using a Q-learning model. Results from Experiment 1 show that subjects who completed the intermixed task paradoxically valued the no-feedback outcome as a reinforcer when it occurred on reinforcement-based trials, and as a punisher when it occurred on punishment-based trials. This is supported by patterns of empirical responding, where subjects showed more win-stay behavior following an explicit reward than following an omission of punishment, and more lose-shift behavior following an explicit punisher than following an omission of reward. In Experiment 2, results showed similar performance whether subjects received reward-based or punishment-based trials first. However, when the Q-learning model was applied to these data, there were differences between subjects in the reward-first and punishment-first conditions on the relative weighting of neutral feedback. Specifically, early training on reward-based trials led to omission of reward being treated as similar to punishment, but prior training on punishment-based trials led to omission of reward being treated more neutrally. This suggests that early training on one type of trials, specifically reward-based trials, can create a bias in how neutral feedback is processed, relative to those receiving early punishment-based training or training that mixes positive and negative outcomes.

## Introduction

Prior research has shown that different rearrangement of task trials affects learning. For example, acquisition of fear conditioning in humans depends on the ordering of presentation of fear (CS+) and safety (CS−) trials (Esteves et al., 1994; Ohman and Soares, 1998; Katkin et al., 2001; Morris et al., 2001; Wiens et al., 2003). In a study by Wiens et al. (Wiens et al., 2003), one group of subjects received CS+ and CS− trials in a random order (differential group) and another group received CS+ and CS− trials in a non-random restricted manner. It was found that skin conductance response to CS+ and CS− was not significantly different in the random condition, but skin conductance responses to CS+ was significantly larger than that to CS− in the restricted condition. Similarly, although the forward and backward blocking paradigms have the same trials, albeit arranged differently, research has shown that the backward blocking effect is weaker than the forward blocking effect (Chapman, 1991; Lovibond et al., 2003). Other studies show that trial order also affect motor learning by observation (Brown et al., 2010). Using a concurrent discrimination task, we have previously found that training subjects to discriminate among a series of pairs of stimuli simultaneously (concurrent condition) takes more trials than learning to discriminate among each pair individually (shaping condition; Shohamy et al., 2006). In short, a number of studies suggest that trial order might impact cognitive performance.

However, the mechanisms underlying these effects remain a matter of debate. Chapman (1991) argues that associative learning models (e.g., Rescorla-Wagner’s 1972 model; Rescorla and Wagner, 1972) and statistical models (e.g., multiple linear regression) cannot account for trial order effects.

Computational models of decision-making are increasingly being used to interpret behavioral results and help understand underlying information-processing mechanisms that could produce individual patterns of behavior (Frank et al., 2007; Dickerson et al., 2011). One class of models, reinforcement learning (RL) models, assumes that trial-and-error learning results in the learner coming to choose actions that are expected to maximize reward and/or minimize punishment. Prediction error (PE), the difference between expected and experienced outcomes, is used to update the learner’s expectations and guide action selection. PE is positive when there is unexpected reward (or an expected punisher fails to occur) and negative when there is unexpected punishment (or an expected reward fails to occur). Learning can be affected by a number of free parameters in RL models, such as *LR+*, the learning rate when PE > 0, *LR−*, the learning rate when PE < 0, and β, an explore/exploit parameter which governs the tendency to repeat previously-successful responses or explore new ones. For each individual subject, values of the free parameters that led the model to display behavior that best mimicked that individual’s observed behavior are identified; differences in the obtained parameters suggest mechanisms underlying different performance as a result of task condition. Previous research has used similar computational models to fit model parameter values for each subject to genetic (Frank et al., 2007), brain imaging (O’Doherty et al., 2003; Dickerson et al., 2011) and patient data (Moustafa et al., 2008; Myers et al., 2013).

In this study, we test the effect of trial ordering on a probabilistic categorization task that involves both reward and punishment-based category learning (Bódi et al., 2009). This task has the feature that reward-based trials, which can result in either reward or no feedback outcomes, are intermixed with punishment-based trials, which can result in either punishment or no feedback outcomes; thus, the no-feedback outcome is ambiguous as it can signal either missed reward (similar to a punishment) or missed punishment (similar to a reward). Prior studies with this task have documented differential learning from reward and punishment in patient populations including medicated and unmedicated patients with Parkinson’s disease (Bódi et al., 2009), major depressive disorder (Herzallah et al., 2013), schizophrenia (Somlai et al., 2011), and symptoms of post-traumatic stress disorder (Myers et al., 2013), as well as individual differences in learning as a function of genetic haplotypes (Kéri et al., 2008) and of personality traits such as novelty seeking (Bódi et al., 2009) and behavioral inhibition (Sheynin et al., 2013). However, the effects of trial order on this task have not heretofore been considered.

Here, in Experiment 1, we started by considering the “standard” task in which reward-based and punishment-based trials are intermixed in each training block. Then, we fit subjects’ behavioral data with a RL model (Watkins and Dayan, 1992; Sutton and Barto, 1998) to investigate mechanisms underlying subjects’ performance. Based on prior computational modeling of this task (Myers et al., 2013), we expected that subjective valuation of the ambiguous no-feedback outcome might vary considerably across subjects. In Experiment 2, we considered a “separated” version of the task, in which subjects are administered reward-based and punishment-based trials in different blocks, and the same model was applied to see how different trial order might affect these mechanisms. We hypothesized that both learning and valuation of the ambiguous no-feedback outcome might differ, depending on whether reward-based or punishment-based training occurred first.

## Methods

### Experiment 1

#### Participants

Experiments 1 and 2 were run concurrently, with participants randomly but evenly assigned to one experimental group. For experiment 1, participants included 36 healthy young adults (college undergraduates, mean age 20.0 years, SD 1.4; 66.7% female). For their participation, subjects received research credit in a psychology class. Procedures conformed to ethical standards laid down in the Declaration of Helsinki for the protection of human subjects. All participants signed statements of informed consent prior to inclusion in the study.

#### Behavioral Task

The task was as previously described (Bódi et al., 2009; Myers et al., 2013) and was conducted on a Macintosh computer, programmed in the SuperCard language (Allegiant Technologies, San Diego, CA, USA). The participant was seated in a quiet testing room at a comfortable viewing distance from the computer. The keyboard was masked except for two keys, labeled “A” and “B” which the participant used to enter responses. A running tally at the bottom of the screen showed the current points accumulated; this tally was initialized to 500 points at the start of the experiment.

**Figure 1:**
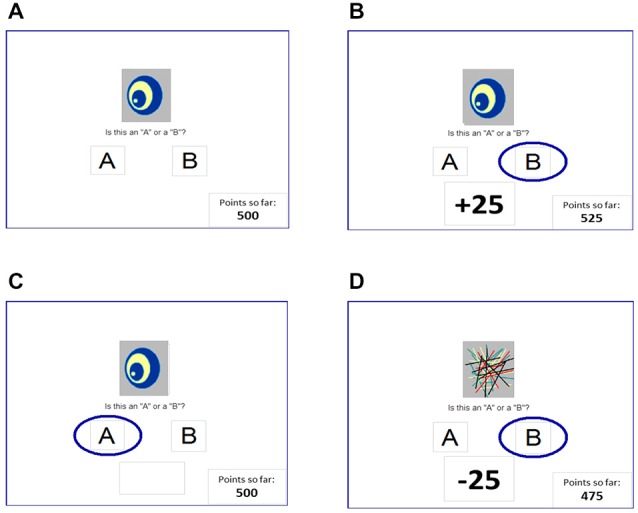
**The reward- and punishment-learning task (Bódi et al., 2009). (A)** On each trial, a stimulus appears and the subject guesses whether this stimulus belongs to category “A” or category “B.” For two stimuli, correct responses are rewarded **(B)** but incorrect responses receive no feedback **(C)**; and for the other two stimuli, incorrect responses are punished **(D)** but correct responses receive no feedback. In Experiment 1, reward-based and punishment-based trials were interleaved, as in the original Bódi et al. (2009) study; in Experiment 2, reward-based and punishment-based trials were presented in separate blocks.

On each trial, participants viewed one of four images, and guessed whether it belonged to category A or category B (Figure 1A). For each participant, the four images were randomly assigned to be stimuli S1, S2, S3, and S4. On any given trial, stimuli S1 and S3 belonged to category A with 80% probability and to category B with 20% probability, while stimuli S2 and S4 belonged to category B with 80% probability and to category A with 20% probability. Stimuli S1 and S2 were used in the reward-learning task. Thus, if the participant correctly guessed category membership on a trial with either of these stimuli, a reward of +25 points was received (Figure 1B); if the participant guessed incorrectly, no feedback appeared (Figure 1C). Stimuli S3 and S4 were used in the punishment-learning task. Thus, if the participant guessed incorrectly on a trial with either of these stimuli, a punishment of –25 was received (Figure 1D); correct guesses received no feedback. Thus, the no-feedback outcome, when it arrived, was ambiguous, as it could signal lack of reward for an incorrect response (if received during a trial with S1 or S2) or lack of punishment for a correct response (if received during a trial with S3 or S4). Participants were not informed which stimuli were to be associated with reward vs. punishment.

At the start of the experiment, the participant saw the following instructions: in this experiment, you will be shown pictures, and you will guess whether those pictures belong to category “A” or category “B”. A picture doesn’t always belong to the same category each time you see it. If you guess correctly, you may win points. If you guess wrong, you may lose points. You’ll see a running total of your points as you play. (We’ll start you off with a few points now.)

The task included a short practice phase, which showed the participant an example of correct and incorrect responses to sample punishment-based and reward-based trials. These practice trials used images other than S1–S4. The practice phase was followed by 160 training trials, divided into four blocks of 40 trials, with each stimulus appearing 10 times per block. Trials were separated by a 2 s interval, during which the screen was blank. At the end of the experiment, if the subjects’ total had fallen below the starting tally of 500 points, additional trials with S1 and S2 were added until the tally reached 525 points; these extra trials were not included in the data analysis.

The probabilistic nature of the task meant that an optimal response across trials (i.e., “A” for S1 and S3; “B” for S2 and S4) might not be correct on a particular trial. Therefore, on each trial, the computer recorded reaction time (in ms) and whether the participant’s response was optimal, regardless of actual outcome (points gained or lost). In addition, for each stimulus, we recorded number of win-stay responses (defined as trials on which the subject repeated a response that had received reward or non-punishment on the prior trial with that stimulus) and number of lose-shift responses (defined as trials on which the subject did not repeat a response that had received punishment or non-reward on the prior trial with that stimulus).

Mixed ANOVA with within-subject factors of block and trial type (reward vs. punishment) and between-subjects factor of gender were used to analyze the data; for analyses of reaction time, response type (optimal vs. non-optimal response) was also included as a between-subjects factor. Levene’s test was used to confirm assumptions of homogeneity of variance. Where Mauchly’s test indicated violations of assumptions of sphericity, Greenhouse-Geisser correction was used to adjust degrees of freedom for computing *p*-values from *F*-values. The threshold for significance was set at α = 0.05 with Bonferroni correction used to protect significance values under multiple comparisons (e.g., *post hoc* testing).

#### Computational Model

Here, we modeled the observed behavioral results using a Q-learning model (Watkins and Dayan, 1992; Sutton and Barto, 1998; Frank et al., 2007) which uses the difference between expected and experienced outcomes to calculate PE which is then used to update predictions and guide action selection. The variant used here is the gain-loss model, which allows separate learning rates when PE is positive-valued or negative-valued (Frank et al., 2007).

Specifically, given stimulus *s*, each possible action *r* (here, choice to categorize the stimulus as “A” or “B”) has a value *Q_r,s_(t)* at trial *t*. All *Q*-values are initialized to 0 at the start of a simulation run (*t* = 0). The *Q*-values are used to determine the probability of choosing each response via a softmax logistic function:

(1) $$\mathrm{Pr}(r="A")=\frac{e^{Q_{A,s}(t)/\beta }}{e^{Q_{A,s}(t)/\beta }+e^{Q_{B,s}(t)/\beta }}$$

(2) Pr(r="B")=1−Pr(r="A")

As described above, *β* reflects the participant’s tendency to either exploit (i.e., to choose the category with the currently highest *Q* value) or explore (i.e., to randomly choose a category).

PE is then computed for that trial based on the subject’s actual response r* and the feedback *R(t)* that the subject received on that trial:

(3) $$PE(t)=R(t)-Q_{r*,s}(t)$$

Here, *R(t)* is +1 for reward feedback, −1 for punishing feedback, and *R0* for the no-feedback outcome. In the prior (Myers et al., 2013) paper, *R0* was a free parameter that could vary between −1 (similar to punishment) and +1 (similar to reward).

Finally, the *Q*-value for the selected stimulus-response pair was updated based on PE:

(4) $$Q_{r*,s}(t+1)=Q_{r*,s}(t)+a*PE$$

Here, *β* is the learning rate, set to *LR+* if PE > 0 and to *LR−* if PE < 0.

First, we considered the model previously applied to data from this task in Myers et al. (2013); this model included four free parameters: *LR+*, *LR−*, *β*, and *R0*. We also considered a five-parameter model in which the value of *R0* could be different on reward-based (*R0rew*) than on punishment-based (*R0pun*) trials, allowing for the possibility that subjects might value the no-feedback outcome differently on these two types of trial. Theoretically optimal performance would be obtained if *R0rew* approached −1 (similar to punishment, and maximally different from *R+*) while *R0pun* approached +1 (similar to reward, and maximally different from *R−*). Simulations (not shown) confirmed that this pattern indeed obtained when the model was run on hypothetical subject data in which optimal responses were always, or nearly always, executed.

Finally, we considered an alternate 4-parameter model in which *R0* was a free parameter by *R0rew* = −1*R0pun, i.e., the value of the no-feedback outcome is equal in magnitude but opposite in valance for the two trial types.^1^

For each of the three models under consideration, values of the free parameters were estimated for each participant, based on that participant’s trial-by-trial choices and the feedback received. To do this, we searched through parameter space, allowing *LR+, LR−* and *β* to vary from 0 to 1 in steps of 0.05 and *R0* to vary from −1 to +1 in steps of 0.1, to find the configuration of parameter values that minimized the negative log likelihood estimate (*negLLE*) across *n* trials:

(5) $$negLLE=-\underset{t=1\ldots n.}{\sum }\log\mathrm{Pr}(r=r*)$$

In plotting results, for clarity of interpretation, this value is transformed into a probability value, *p(choice) = exp(-negLLE/n)*, where *p(choice)* = 0.5 means chance and *p(choice)* = 1 means perfect replication of subject data.

To compare the three models, we used the Akaike Information Criterion (AIC; Akaike, 1974), which compares goodness-of-fit in terms of minimal *negLLE* while penalizing models that have more free parameters: *AIC* = 2*negLLE + 2*k, where *k* is the number of free parameters. We also used the Bayesian Information Criterion (BIC; Schwartz, 1978) which additionally considers number of subjects:

(6) BIC=2*negLLE+k*ln(x)

where *x* is the number of trials. Note that BIC assumes that one of the models being compared is the correct model, which is an assumption that is not necessarily provable for this type of dataset, while AIC only assesses which of the models is most efficient at describing the data while not necessarily assuming any are probably correct.

In addition to evaluating the three models described above, we also considered several additional variants: a three-parameter model where *R0* was held constant at 0 (leaving *LR+, LR−*, and *β* free to vary), a two-parameter model where *LR+* and *LR−* are constrained to be the same value, as in a standard Q-learning model (leaving only a single *LR* and *β* free to vary), and models where *R0* (singly, or separately for *R0rew* and *R0pun*) were free to vary but the other parameters were fixed using mean values derived from the five-parameter value; none of these other variants performed as well as the four- and five-parameter models, and for conciseness results with these variants are not described further here.

Further, to compare models we used the random effects Bayesian model selection procedure described in Stephan et al. (2009) and Penny et al. (2010), which takes into account the possibility that different models may have generated different subjects’ data. Based on prior studies, we consider one model the winning model when protected exceedance probability for that model is larger than 0.9.

## Results

### Behavioral Task

**Figure 2:**
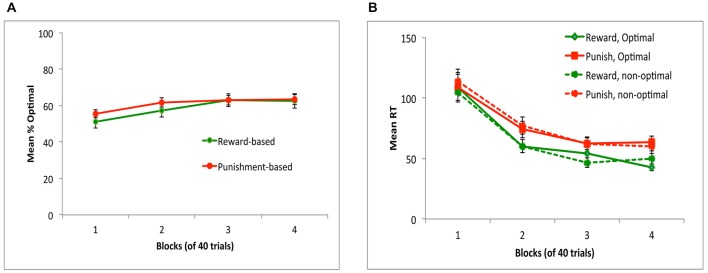
**Performance on the task in Experiment 1. (A)** Subjects performed equally well on reward-based and punishment-based trials, assessed as percent optimal responding. **(B)** Mean reaction time (RT) in milliseconds decreased across training blocks, and was slower on punishment-based than reward-based trials. RT did not differ on trials where subjects gave the optimal (correct) vs. non-optimal (incorrect) response.

Figure 2A shows performance on reward-based and punishment-based trials, over the four blocks of the experiment. There was significant learning, indicated by a within-subjects effect of block (*F*_(2,47,84.12)_ = 6.22, *p* = 0.002) with no effect of trial type or sex and no interactions (all *p* > 0.200), indicating that average learning accuracy was similar across reward-based and punishment-based trials. Figure 2B shows reaction times (RT) for optimal and non-optimal responses on each trial type, over the course of the experiment. While all subjects made at least one optimal response to each trial type in each block, eight subjects did not make any non-optimal responses to at least one trial type in at least one block, meaning average RT could not be computed. Rather than dropping these eight subjects from analysis, we did separate rmANOVA of RT on optimal responses (calculated for all 36 subjects) and on non-optimal responses (for those 28 subjects who made at least one non-optimal response on each trial type in each block); Bonferroni correction was used to adjust alpha to 0.025 to protect significance levels under multiple tests. For optimal responses, there was a significant decrease in RT over blocks (*F*_(1.75,59.45)_ = 21.92, *p* < 0.001) as well as a main effect of trial type, with RT slower on punishment-based than reward-based trials (*F*_(1,34)_ = 26.61, *p* < 0.001). For non-optimal responses, the same pattern was observed: a significant decrease in RT over blocks (*F*_(1.85,48.02)_ = 34.97, *p* < 0.001) and significantly slower responding on punishment-based than reward-based trials (*F*_(1,26)_ = 8.48, *p* = 0.007). However, the interaction between block and trial type and all effects and interactions involving gender did not reach corrected significance.

**Figure 3:**
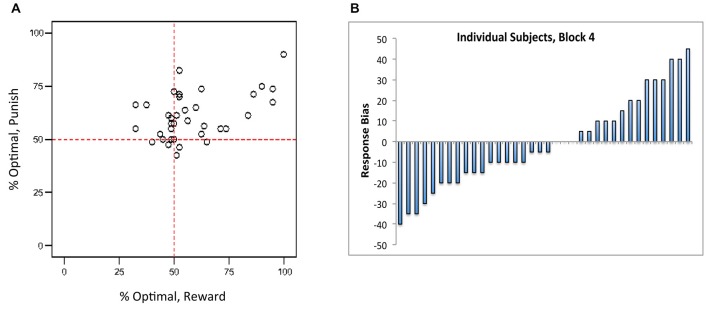
**(A)** Individual subjects showed considerable individual variation in reward-based and punishment-based learning, plotted here in terms of percent optimal responding on each trial type. **(B)** Near the end of the experiment (block 4), individual subjects showed a “bias” (difference between performance on reward-based vs. punishment-based trials) that varied from strongly negative (better performance on punishment-based trials) to strongly positive (better performance on reward-based trials). Each bar represents an individual subject, ordered along the *x*-axis by bias.

However, Figure 3A shows that there was considerable individual variability in performance on reward-based and punishment-based trials, with many subjects performing considerably better on one type of trial than another. Following Sheynin et al. (2013), we considered a “bias” measurement, defined as the difference between a subject’s performance on reward-based trials and on punishment-based trials in the final training block; thus, a negative bias indicates better performance on punishment-based trials, a positive bias indicates better performance on reward-based trials, and a bias of 0 indicates equally good performance on both types of trial. Figure 3B shows that, although bias was near 0 when averaged across subjects, many individual subjects showed a bias for either reward- or punishment-based trials that persisted through block 4 of the experiment.

**Figure 4:**
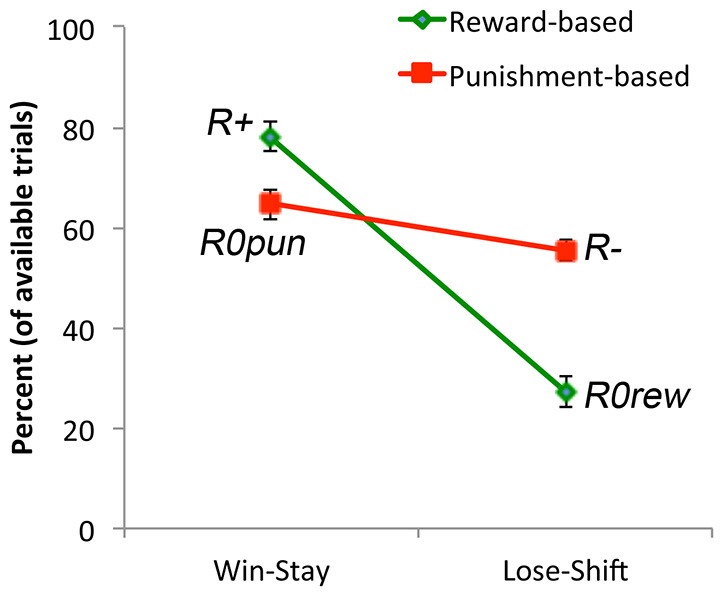
**Win-stay occurs when subjects make a response to a stimulus and receive reward (or non-punishment) and then repeat the same response on the next trial with that stimulus**. Subjects exhibited more win-stay responses following an explicit reward (R+) than following a non-punishment (*R0pun*). Lose-shift occurs when subjects make a response to a stimulus and receive punishment (or non-reward) and then make a different response on the next trial with that stimulus. Subjects exhibited more lose-shift responses following an explicit punishment (R−) than following a non-reward (*R0rew*).

Finally, we examined win-stay and lose-shift behavior. It would be expected that subjects would generally show win-stay after an explicit reward, and generally show lose-shift after an explicit punishment (although, due to the probabilistic nature of the task, not every punishment should trigger abandonment of a response rule). If the no-feedback outcome were treated as similar to a punisher on reward-based trials, then it should also trigger lose-shift; conversely, if the no-feedback outcome were treated as similar to a reward on punishment-based trials, then there it should also trigger win-stay. However, Figure 4 shows that, on average, subjects exhibited more win-stay responses on reward-based than punishment-based trials, and more lose-shift responses on punishment-based than reward-based trials. Mixed ANOVA confirmed these impressions: there was a main effect of response, with subjects producing more win-stay than lose-shift responses overall (*F*_(1,34)_ = 43.93, *p* < 0.001), as well as a main effect of trial type (*F*_(1,34)_ = 18.73, *p* < 0.001), and an interaction (*F*_(1,34)_ = 101.96, *p* < 0.001). *Post hoc* pairwise *t*-tests to examine the interaction, with alpha adjusted to 0.025, revealed significantly more win-stay behavior on reward-based than-punishment-based trials (*t*_(35)_ = 8.22, *p* < 0.001) but significantly more lose-shift behavior on punishment-based than reward-based trials (*t*_(35)_ = 7.60, *p* < 0.001). The omnibus ANOVA also revealed a main effect of sex, with males generally exhibiting more win-stay and lose-shift behaviors than females (*F*_(1,34)_ = 4.83, *p* = 0.035), and a three-way interaction between response type, trial type, and gender (*F*_(1,34)_ = 5.36, *p* = 0.027); however, none of the specific comparisons in the interaction reached significance on *post hoc* testing.

### Computational Model

Prior work with this task in a different population (veterans with and without severe PTSD symptoms) led us to note that an important source of individual differences might be variability in how people assigned reinforcement value to the ambiguous no-feedback outcome (Myers et al., 2013). The individual differences observed in this experiment, together with the finding that win-stay occurred significantly more often following a reward than a no-punishment outcome, while lose-shift occurred significantly more often following a punishment than a no-reward outcome, led us to consider whether individual differences in valuation of the ambiguous outcome might similarly underlie the behavior observed in the current study.

Following the earlier Myers et al. (2013) paper, we considered an RL model with four free parameters, the learning rates *LR+* and *LR−*, the “temperature” parameter *β*, and the reinforcement value of the no-feedback outcome *R0*. We also considered a more elaborate five-parameter model, where the no-feedback outcome could be valued differently when it occurred on a reward-learning trial (*R0rew*, signaling failure to obtain reward) and when it occurred on a punishment-learning trial (*R0pun*, signaling successful avoidance of punishment). We also considered a second four-parameter model where *R0rew* was free to vary while *R0pun* was set at −1*R0rew.

All models generated unique combinations of best-fit parameters for every subject with the exception of the five-parameter model, which generated multiple sets of best-fit parameters. In each of these cases, there were unique best-fit values for all estimated parameters except *R0rew*; however, for two subjects, the five-parameter model produced equally low *negLLE* for any value of *R0rew* >= 0 (given best-fit values for the remaining parameters), and for one subject, the model produced equally low *negLLE* for any value of *R0rew* <= 0 (given best-fit values for the remaining parameters). For subsequent analyses, the neutral value of *R0rew* = 0 was assigned as the best-fit parameter for these three subjects.

**Figure 5:**
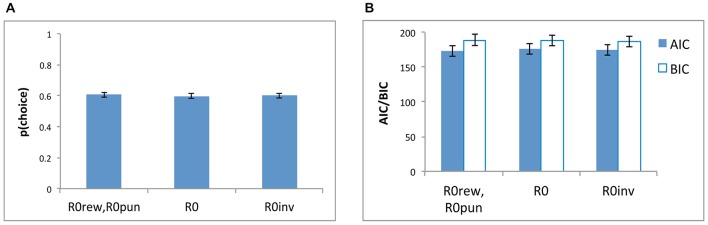
Model comparisons based on (A) *p(choice)* which is derived from *negLLE* (0.5 indicating chance and 1.0 indicating perfect fit), and (B) AIC and BIC, for the three models: the five-parameter model with where both *R0rew* and *R0pun* were free parameters (“*R0rew*, *R0pun*”), a simpler four-parameter model (“*R0*”) with a single free parameter for *R0* (where *R0rew* = *R0pun*), and a four-parameter model (“*R0inv*”) where *R0rew* = −1*R0pun.

Figure 5 shows that the models were similar in their ability to reproduce the data, both in terms of *negLLE* (Figure 5A) and also in terms of *AIC* and *BIC* (Figure 5B).

**Figure 6:**
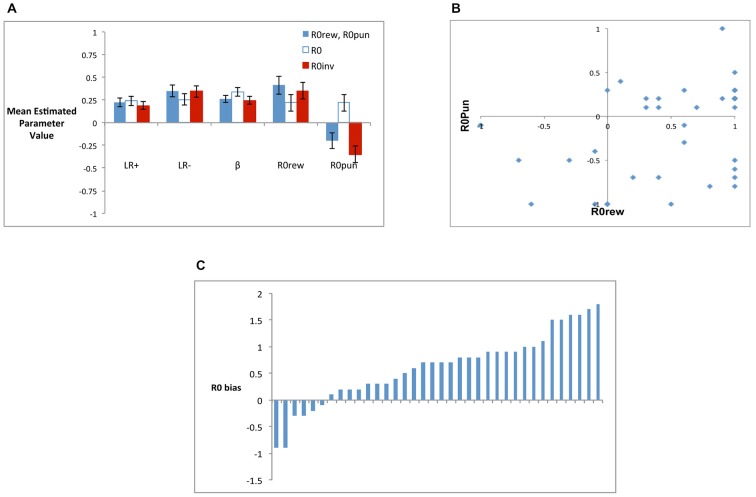
**(A)** Mean estimated parameter values for the three models, including the five-parameter model (*R0rew*, *R0pun*), the *R0* model (where *R0rew* = *R0pun*) and the *R0inv* model (where *R0rew* = −*R0pun*). **(B)** Individual subjects had considerable variability in best-fit values of *R0rew* and *R0pun*, and no subjects were best-fit by a combination including the “optimal” pattern of both *R0rew* < 0 (similar to punishment) and *R0pun* > 0 (similar to reward). **(C)** *R0* bias for individual subjects, defined as the difference in best-fit value of *R0rew* and *R0pun*; bars represent individual subjects arbitrarily ordered by increasing values of *R0* bias.

Figure 6A shows mean estimated parameter values for the three models; although *R0rew* and *R0pun* are plotted separately, note that *R0rew = R0pun* for the *R0* model, while *R0rew* = −1*R0pun for the *R0inv* model. While all models had similar estimated mean values for *LR+* (rmANOVA, *F*_(1.52,53.21)_ = 1.21, *p* = 0.297) and *LR−* (*F*_(1.34,46.86)_ = 3.22, *p* = 0.068), there were differences on estimated values of *β* (*F*_(1.22,42.66)_ = 6.88, *p* = 0.008), which was significantly larger in the *R0* model than in the five-parameter (*t*_(35)_ = 2.48, *p* = 0.018) or *R0inv* models (*t*_(35)_ = 2.86, *p* = 0.007), which did not differ (*t*_(35)_ = 1.47, *p* = 0.151). Finally, the largest differences between models were observed in estimated values of *R0rew* and *R0pun*. Specifically, as shown in Figure 6A, the five-parameter model produced a mean value of *R0rew* that was greater than 0, and a mean value of *R0pun* that was less than 0. This pattern was echoed in the *R0inv* model but not in the *R0* model, where both *R0rew* and *R0pun* were constrained to be equal, resulting in a weakly positive value for both. rmANOVA confirmed that estimated values of *R0rew* did not differ across the three models (*F*_(1.48,51.00)_ = 1.56, *p* = 0.217) but values of *R0pun* did (*F*_(1.62,56.57)_ = 16.25, *p* < 0.001). Specifically, the value of *R0pun* in the *R0* model was significantly greater than in the five-parameter or *R0inv* models (all *t* > 4.5, all *p* < 0.001), but the value in the latter two models did not differ (*t*_(35)_ = 1.65, *p* = 0.108).

Based on these analyses of mean scores, the *R0inv* model was a closer approximation to the five-parameter model than the *R0* model. However, Figure 6B shows that there was considerable individual variability in values of *R0rew* and *R0pun* in the five-parameter model, such that mean values may not adequately capture the qualitative patterns in the data. Specifically, while the *R0* model constrained subjects to have equal values for *R0rew* and *R0pun*, and the *R0inv* model constrained them to be opposite in valence, Figure 6B shows that neither constraint adequately described the values generated by the five-parameter model. Rather, while a majority of subjects had estimated values of *R0rew* > 0 and *R0pun* < 0 (as also indicated in Figure 6A), some individual subjects assigned the same valence to these parameters while others did not. Interestingly, for no subject was the theoretically optimal pattern (*R0rew* < 0 and *R0pun* > 0) observed. There were also differences in the relative magnitude of *R0rew* and *R0pun*. Figure 6C shows *R0* bias, defined as the difference between estimated values of *R0rew* and *R0pun*, for individual subjects. While only 2 of 36 subjects (5.6%) had *R0* bias < −0.5, 22 of 36 subjects (61.1%) had *R0* bias > = +0.5.

**Figure 7:**
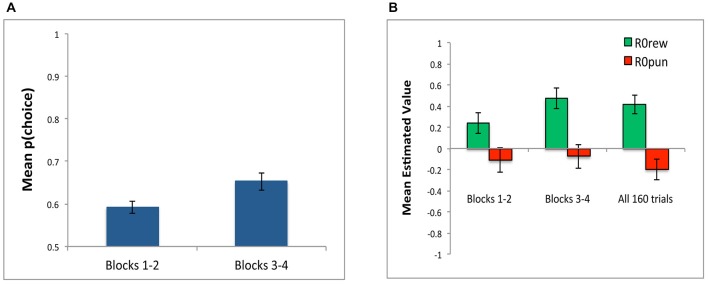
**Parameter estimates in Experiment 1 for Blocks 1 and 2 (first 80 trials) and Blocks 3 and 4 (last 80 trials). (A)** Model fit, plotted as *p(choice)* is better in last 80 trials compared to first 80 trials, reflecting greater consistency in subject responding as training progressed. **(B)** The value of estimated parameters *R0rew* and *R0pun*, showing mean values of *R0rew* > 0 and *R0pun* < 0, whether assessed over the first 80 trials (Blocks 1–2), last 80 trials (Blocks 3–4), or entire experiment (all 160 trials).

In addition to conducting simulations over all 160 training trials, we also conducted separate simulations to determine best-fit parameters over the first two blocks (first 80 trials) and over the last two blocks (last 80 trials). As shown in Figure 7A, model fit was better (lower *negLLE*, reflected in higher *p(choice)*) when the model was fit to blocks 3 and 4; this is unsurprising since subjects should have developed more consistent response rules later in training. As shown in Figure 7B, the value of estimated parameters *R0rew* and *R0pun* show the same qualitative pattern of *R0rew* > 0 and *R0pun* < 0 in the early blocks of training, and also in the later blocks of training, as when the model is applied to all 160 trials.

Importantly, random effects Bayesian model selection comparing the five-parameter model with the two four-parameter models indicated strong evidence in favor of the five-parameter model, with posterior probability for this model calculated as *r* = 0.63, compared with *r* = 0.11, −0.26 for each of the smaller models. Further, we compared protected exceedance probabilities among the three models as well as among each two models separately. We found that comparing all three models at once yields exceedance probabilities of 0.9998, 0.0001 and 00001 for five-parameter model, standard four-parameter-model, and four-parameter-model in which *R0* was a free parameter by *R0rew* = −1*R0pun, respectively.

## Experiment 2

Experiment 1 was examined here as a standard baseline task for reward and punishment learning, as used in prior studies (Bódi et al., 2009; Kéri et al., 2010; Myers et al., 2013; Sheynin et al., 2013). Because reward-based and punishment-based trials were intermixed in Experiment 1, the no-feedback outcome was ambiguous. The central finding of the modeling was that—contrary to what might be defined as “optimal” behavior, subjects tended to value the ambiguous feedback as positive (similar to reward) on reward-based trials, and as negative (similar to punishment) on punishment-based trials.

In Experiment 2, we use a separated task design in which reward and punishment trials are conducted separately, in different training blocks. The no-feedback outcome is arguably unambiguous here, since in a block of reward-based trials it always signals missed reward (similar to a punishment) while in a block of punishment-based trials it always signals missed punishment (similar to reward). We here predicted that estimated values of *R0* might differ accordingly both early in training, while subjects were experiencing only a single trial type, as well as later in training, as a function of early learning.

### Participants

Participants were drawn from the same population as Experiment 1 and included 36 healthy young adults (college undergraduates, mean age 19.6 years, SD 1.6; 63.9% female). As in Experiment 1, participants received research credit in a psychology class. Procedures conformed to ethical standards laid down in the Declaration of Helsinki for the protection of human subjects. All participants signed statements of informed consent prior to inclusion in the study.

### Behavioral Task

The task was the same as in Experiment 1 except that subjects were randomly assigned to either a Reward-First (*n* = 17) or Punish-First (*n* = 19) condition. For those in the Reward-First condition, all 40 reward-based trials (stimuli S1 and S2) appeared in blocks 1 and 2 while all 40 punishment-based trials (stimuli S3 and S4) appeared in blocks 3 and 4. For the Punish-First condition this order was reversed. Thus, the no-feedback outcome was no longer ambiguous, as it consistently signaled lack of reward during the reward-learning blocks and consistently signaled lack of punishment during the punishment-learning trials. Subjects were not informed that trials were blocked by type, nor were subjects explicitly signaled of the shift between blocks 2 and 3.

### Computational Model

The same five-parameter model described in Experiment 1 above was applied to the data from this experiment. In addition to calculating best-fit parameters based on the data from the complete set of 160 trials, we also applied the models to just the first 80 trials (blocks 1 and 2) and just the last 80 trials (blocks 3 and 4), when subjects were learning either the reward-based or punishment-based task.

## Results

### Behavioral Task

**Figure 8:**
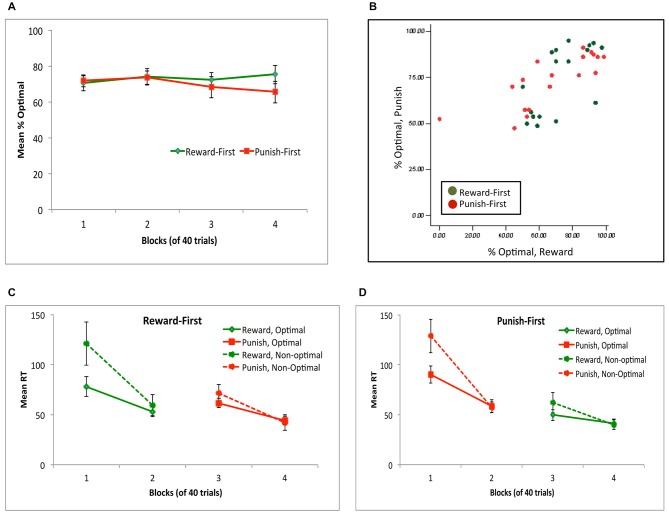
**(A)** Performance across the four blocks, for subjects in the Reward-First and Punish-First conditions of Experiment 2. **(B)** Individual subject data, plotted as percent optimal responding on reward-based and punishment-based trials. **(C,D)** Mean reaction time (RT) in ms for subjects in the Reward-First **(C)** and Punish-First **(D)** conditions, shown separately for trials where the optimal and non-optimal response was given.

Figure 8A shows performance across all four blocks, for subjects assigned to the Reward-first and Punish-first conditions. Mixed ANOVA confirmed no significant change in performance across the four blocks (*F*_(2.22,75.42)_ = 0.70, *p* = 0.553), no effect of condition (*F*_(1,34)_ = 0.31, *p* = 0.579) and no interaction (*F*_(2.22,75.42)_ = 1.55, *p* = 0.208). Figure 8B shows individual subject performance on the reward-based and punishment-based trials, and again shows considerable individual variation on performance to the two trial types for subjects in either experimental condition.

Figures 8C,D show mean RT for subjects in each condition. Again, not all subjects made all response types on every block; for example, four subjects (two in each condition) made no non-optimal responses in block 4. Thus, as in Experiment 1, separate mixed ANOVAs of RT were conducted on optimal and non-optimal responses, with alpha adjusted to 0.025 to protect significance. For optimal responses, there was a significant effect of block (*F*_(2.09,69.09)_ = 25.85, *p* < 0.001) but no effect of condition (*F*_(1,33)_ = 0.02, *p* = 0.904) and no interaction (*F*_(2.09,69.09)_ = 2.29, *p* = 0.107). For non-optimal responses, the pattern was similar: a within-subjects effect of block (*F*_(1.37,38.35)_ = 20.29, *p* < 0.001) but no effect of condition (*F*_(1,28)_ = 0.14, *p* = 0.714) and no interaction (*F*_(1.37,38.35)_ = 0.23, *p* = 0.708). Specifically, for both optimal and non-optimal responses, RT decreased from block 1 to block 2 (all *t* > 5, all *p* < 0.001) and from block 3 to block 4 (all *t* > 4, all *p* < 0.001) but did not change when the trial types were shifted from block 2 to 3 (all *t* < 1.5, all *p* > 0.100).

**Figure 9:**
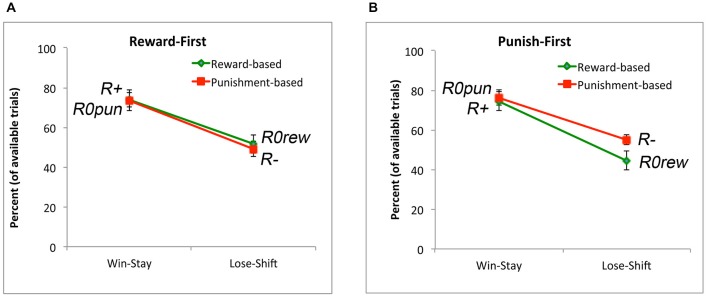
**Win-stay and lose-shift behavior for subjects in the (A) Reward-First and (B) Punish-First conditions**. In both conditions, there was more win-stay than lose-shift behavior for both reward and punishment trials, but (unlike Experiment 1) there was no difference in win-stay behavior to explicit reward vs. non-punishment, or in lose-stay behavior to explicit punishment vs. non-reward.

Finally, we again examined win-stay and lose-shift behavior. As suggested by Figure 9, there was no main effect of condition (*F*_(1,34)_ = 0.04, *p* = 0.842); however, subjects exhibited more win-stay than lose-shift behavior (*F*_(1,34)_ = 27.69, *p* < 0.001). There was also an interaction between trial type and experimental condition (*F*_(1,34)_ = 9.41, *p* = 0.004); however, no *post hoc* comparisons to explore this interaction survived corrected significance. Thus, the pattern seen in Experiment 1 (Figure 4), where there was more win-stay behavior on reward-based than punishment-based trials, and more lose-shift behavior on punishment-based than reward-based trials, was not observed here.

### Computational Model

Given that the analyses in Experiment 1 identified the five-parameter model as providing lowest *negLLE*, with comparable *AIC* or *BIC* to simpler models, we applied the same five-parameter model here to data from the two experimental conditions. Again, we ran simulations both to fit data from all 160 trials, as well as running additional simulations based on data from just the first 80 or last 80 trials, while subjects were experiencing only a single trial type. Results from both sets of simulations are reported here.

**Figure 10:**
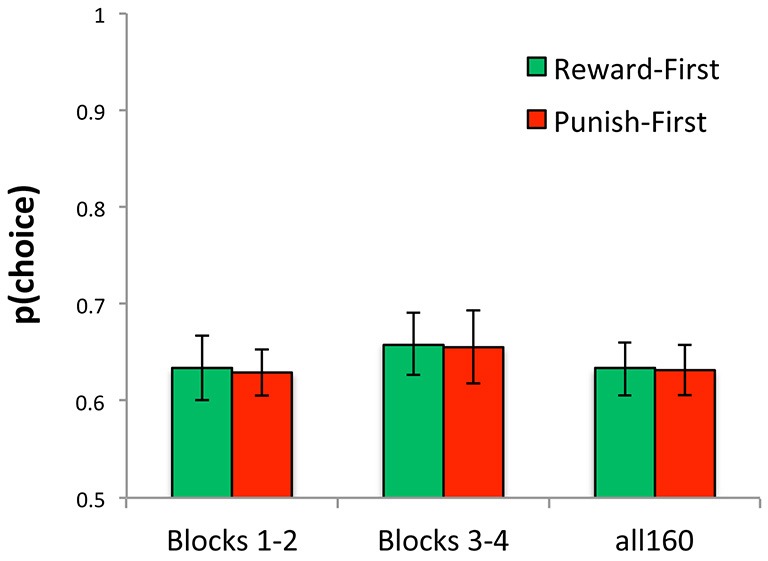
**Ability of model to reproduce the data, plotted here as *p(choice)* derived from *negLLE*, was similar for Reward-First and Punish-First conditions, whether assessed over just the first 80 trials (blocks 1 and 2), just the last 80 trials (blocks 3 and 4), or over all 160 trials**.

Across just the first 80 trials (blocks 1 and 2), the model fit data from the Reward-First and Punish-First conditions equally well (*t*_(34)_ = 0.03, *p* = 0.977); similarly, across all 160 trials there was no difference in *negLLE* between the two conditions (*t*_(34)_ < 0.01, *p* > 0.99). Figure 10 shows these data, rescaled as *p(choice)* which is normalized for number of trials and so can be compared across calculations based on 80 vs.160 trials.

**Figure 11:**
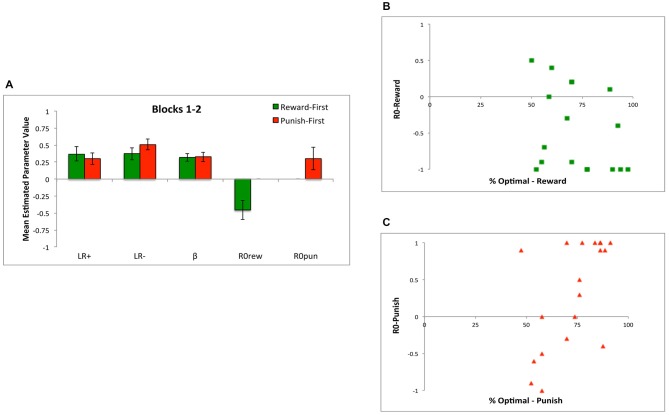
**The five-parameter model applied to data from the first two blocks of Experiment 2. (A)** Applied to the first 80 trials, during which subjects only experienced one trial type, there were no differences between conditions in mean estimated value of *LR+*, *LR−*, or *β*; however, *R0rew* < 0 (similar to a punisher) for subjects in the Reward-First condition, while *R0pun* > 0 (similar to a reward) for subjects in the Punish-First condition. **(B)** Looking at individual data, for subjects in the Reward-First condition, who only experienced reward-based trials over the first 80 trials, there was no significant correlation between estimated values of *R0rew* and performance; **(C)** but for subjects in the Punish-First condition, higher (more positive) values of *R0pun* were associated with better performance.

At the end of the first 80 trials (blocks 1 and 2), there were no differences in the value of *LR+*, *LR−*, or *β* between Reward-First and Punish-First conditions (Figure 11A; *t*-tests, all *t* < 1.5, all *p* > 0.100). Because subjects in each condition had only experienced one type of trial, those in the Reward-First condition had an estimated value for *R0rew* but not *R0pun* (which they had never experienced), while those in the Punish-First condition had an estimated value for *R0pun* but not *R0rew*. As might be expected, for the former group, *R0rew* < 0 (one-sample *t*_(16)_ = 3.29, *p* = 0.005), indicating the no-feedback outcome was valued as similar to a punishment (missed reward); however, for the latter group, *R0pun* was not significantly different from 0 (one-sample *t*_(18)_ = 1.82, *p* = 0.086).

For subjects in the Punish-First condition, there was a strong correlation between the value of *R0pun* over the first 80 trials and performance over those same trials (*r* = 0.603, *p* = 0.006), indicating that those subjects who assigned *R0pun* a more positive value performed better at avoiding the actual punisher in preference to the no-feedback outcome (Figure 11C). However, for subjects in the Reward-First condition, the correlation between *R0rew* and performance was not significant (*r* = −0.337, *p* = 0.185); as shown in Figure 11B, many subjects who valued *R0rew* close to −1 nevertheless performed near chance on the reward-learning trials.

**Figure 12:**
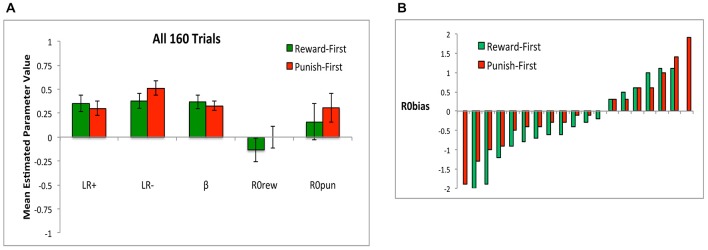
**(A)** When the model was applied to all 16 trials, there were no differences in mean estimated parameter values between conditions, and neither *R0rew* nor *R0pun* differed significantly from 0 in either condition. **(B)** *R0* bias, defined as difference in estimated value between *R0rew* and *R0pun*, for both Reward-First and Punish-First groups (Note there are two more subjects in the Punish-First than the Reward-First condition).

When applying the model to data from all 160 trials, during which all subjects had experienced the same number of both trial types, there were no differences in the value of any estimated parameter between Reward-First and Punish-First conditions (Figure 12A; *t*-tests, all *t* < 1.5, all *p* > 0.100). Here, neither *R0rew* nor *R0pun* differed significantly from 0 in either condition (all *p* > 0.200). Figure 12B shows that the separated task design did qualitatively shift *R0* bias: whereas in Experiment 1, only 2 of 36 subjects (5.6%) had *R0* bias < −0.5 (Figure 6C), here 8 of 17 subjects (47.1%) in the Reward First condition and 5 of 19 subjects (26.3%) in the Punish First condition had *R0* bias <= −0.5. The distribution did not differ across Reward-First and Punish-First conditions (Yates-corrected chi-square test, *χ*^2^ = 0.90, *p* = 0.344).

**Figure 13:**
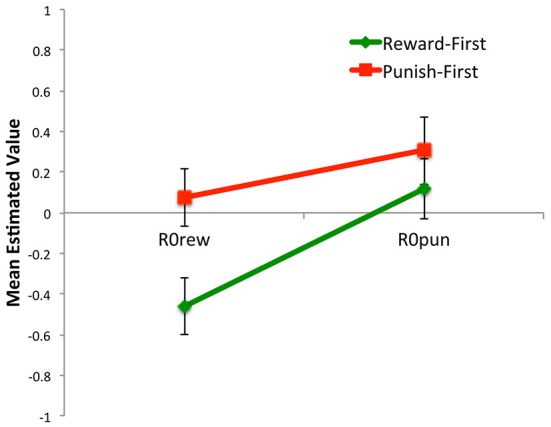
**The value of estimated parameters *R0rew* and *R0pun* for subjects in Reward-First and Punishment-First conditions when the model was applied separately to the first 80 and last 80 trials (i.e., comparing first task learned vs. second task learned)**.

Finally, it can be argued that the first 80 and last 80 trials represent two separate tasks, and so rather than fitting the model to all 160 trials, it is reasonable to fit it once to the first 80 trials and again to the last 80 trials. When the model was fit to just the last two blocks (last 80 trials), there were no differences between conditions on any estimated parameter (Figure 13; all *p* > 0.05), and neither the estimated value of *R0rew* in the Punish-First group (who was now experiencing reward-based trials) nor the estimated value of *R0pun* in the Reward-First group (who was now experiencing punishment-based trials) differed significantly from 0 (all *p* > 0.05).

Thus, comparing the first task (Figure 11A) to the last task (Figure 13) learned, there were effects of task order. Specifically, the estimated value of *R0rew* was greater when reward trials occurred after punishment trials (i.e., in the Punish-First group) than when they were trained without prior experience (i.e., in the Reward-First group; Figure 13; *t*-test, *p* = 0.011). Such differences were not evident in estimated values of *R0pun*—i.e., values were similar whether or not punishment training occurred in naïve subjects, or in subjects who had already experienced reward-based training (*t*-test, *p* = 0.411). No other parameters changed significantly within or across conditions from the first two blocks to the last two blocks (all *p* > 0.08). Thus, training order had a significant effect on *R0rew* but not *R0pun* or any other parameter.

## Discussion

In the current study, we found the following. First, when we applied Q-learning model to the “standard” (intermixed) version of the task in Experiment 1, we found that the five-parameter model weighted the no-feedback outcome differently when it appeared on reward-based trials (*R0rew*, the alternative to explicit reward) than when it appeared on punishment-based trials (*R0pun*, the alternative to explicit punishment). Contrary to what one might think (and, in fact, contrary to what would be “optimal”), subjects tended to value *R0rew* > 0 and *R0pun* < 0. That is, the no-feedback outcome on a reward trial was valued similar to a small reward, while the no-feedback outcome on a punish trial was valued similar to a small punishment. This pattern was similar whether the model was applied to data from all 160 trials or separately to the first 80 trials (blocks 1 and 2) and the second 80 trials (blocks 3 and 4). This suggests that, rather than treating the no-feedback outcome as a contrast to explicit reward, subjects instead tended to value it based on trial type: positively on trials where reward was available, and negatively on trials where punishment might occur.

Second, when we looked at individual subject data from Experiment 1, there was no correlation between estimated values of *R0rew* and *R0pun*; that is, while the group valued these inversely on average, individual subjects did not. One implication of this is that, although we explored several simpler RL models, these generally did not adequately capture the qualitative range of solutions found to describe individual subjects. We also found that individual subjects tended to have *R0* bias > 0, indicating a greater absolute value of *R0rew* than *R0pun*. This would potentially produce somewhat better learning on punish than reward trials in the intermixed group, since a strong positive value of *R0rew* means that the actual reward might only be viewed as marginally more reinforcing than the no-feedback outcome. Such a trend is visible in Figure 1A, although it was not significant here. Prior work with this task, however, has often shown slightly better learning on punishment trials than reward trials in control groups given intermixed training (e.g., Somlai et al., 2011; Myers et al., 2013; Sheynin et al., 2013), although like in the current study this difference does not always reach significance. Such a bias to learn from punishment more readily than from reward is of course consistent with loss aversion theory (Kahneman and Tversky, 2000), which essentially states that losses are psychologically more powerful than gains. We also found that males show more win-stay and lose-shift behaviors than females in this task. The win-stay behavior in our data is similar to other findings from rat studies, although the lose-shift data are different (van den Bos et al., 2012).

The curious finding that subjects in Experiment 1 valued *R0rew* similar to a reward and *R0pun* similar to a punishment warrants further investigation. As a first step, in Experiment 2, we examined subject behavior when reward-based trials were trained first vs. when punishment-based trials were trained first. Visual comparison of learning curves from Experiment 1 (Figure 2A) and Experiment 2 (Figure 8A) shows somewhat better learning in the latter, as might be expected given that the separated conditions of Experiment 2 involve reduced working memory load (only two trial types trained at any given time) and reduced ambiguity of the no-feedback outcome (only one meaning within any given block of trials). However, we found no significant difference on behavioral performance (in terms of percent optimal responding or reaction time) whether the reward-based or punishment-based trials were trained first. This contrasts observations in other tasks showing an effect of trial ordering on behavior (Esteves et al., 1994; Ohman and Soares, 1998; Katkin et al., 2001; Morris et al., 2001; Lovibond et al., 2003; Wiens et al., 2003).

There was, however, an effect on win-stay and lose-shift behavior. Specifically, as shown by Figure 9, there was no main effect of whether reward-based or punishment-based trials were trained first, but *post hoc* tests found no significant difference in win-stay responding on reward trials (where subjects obtained explicit reward) vs. non-punishment trials (where subjects received *R0rew*), and no significant difference in lose-shift responding on punishment trials (where subjects obtained explicit punishment) vs. non-reward trials (where subjects received *R0pun*). This suggests that subjects were treating *R0rew* similar to punishment (missed reward) and *R0pun* similar to reward (missed punishment), in contrast to the results from Experiment 1.

This pattern was echoed when the Q-learning model was applied to data from the first 80 blocks of Experiment 2, when subjects were experiencing either reward-based or punishment-based trials. Specifically, and as might be expected, subjects in the Reward-First condition had estimated values of *R0rew* below 0, indicating that the no-feedback outcome was treated similar to a punishment (missed opportunity for reward). In the Punish-First condition, estimated values of *R0pun* were numerically, but not significantly, greater than 0.

It might have been expected that “switching” tasks in the second half of Experiment 2 might result in the Punish-First group (who was now experiencing only reward-based trials) might similarly develop a positively-valued *R0rew*, while the Reward-First group (who was now experiencing only punishment-based trials) might develop a positively-valued *R0pun*. But this was not the case. Specifically, the estimated value of *R0rew* was greater (closer to 0) when reward-based trials occurred after punishment-based trials (as in the Punish-First group) than when they occurred in a naïve subject (as in the Reward-First group). This suggests that non-reward is valued more negatively in subjects who have only ever experienced reward, compared to subjects who have previously experienced explicit punishment. By contrast, prior exposure to explicit reward did not affect valuation of non-punishment. Thus, training order had a significant effect on *R0rew*, but not *R0pun* or any other estimated parameter.

Finally, when the Q-learning model was applied to data from all 160 trials in Experiment 2, there were no differences in values of estimated parameters for subjects from the Reward-First or Punish-First conditions, and in neither case did estimated values of *R0rew* or *R0pun* differ significantly from 0. Again, the chief contrast with Experiment 1, where trial types were intermixed, appears to be in valuation of *R0rew*, which was strongly positive following intermixed training, but more neutrally-valued after separated training.

The negatively-valued estimates of *R0* in the Reward-First condition are potentially interesting because they are reminiscent of those observed by Myers et al. (2013) in a prior study of veterans with symptoms of post-traumatic stress disorder (PTSD), a disorder that includes pathological avoidance among its defining symptoms. In that earlier study, in which all subjects received intermixed reward and punishment trials, control subjects (with few or no PTSD symptoms) had estimated values of *R0* near +0.5, slightly larger than those obtained in the intermixed group of the current study. By contrast, subjects with severe PTSD symptoms had significantly lower (but still positive) values of *R0*, and those with severe PTSD symptoms who were not receiving psychoactive medication for their symptoms had estimated values of *R0* near 0, similar to that observed in the Reward-First condition of Experiment 2 here. In the current study, we did not assess PTSD symptoms, so we cannot definitively rule out the possibility that PTSD symptoms contributed to the current pattern of results; however, it seems unlikely that severe PTSD would occur at high rates in the college population from which our sample was drawn, nor that such cases if they existed would have been disproportionately assigned to the Reward-First condition.

An alternate hypothesis is that prior training on reward-based trials only created a bias to view neutral outcomes as negatively-valenced, although this bias could be partly remediated by later exposure to punishment-based trials. As current therapy for PTSD often focuses on providing positive and/or neutral feedback, it may be possible that alternate approaches, which explicitly contrast neutral and negative feedback, might be more successful in helping these individuals to reframe their interpretation of neutral outcomes. However, future work should confirm or disconfirm these speculations.

Another relevant prior study has suggested that subjects’ RT depend on the rate of experienced reward, possibly reflecting tonic levels of dopamine (Guitart-Masip et al., 2015). This study differed from ours in many ways: specifically, it was an oddball detection task, with subjects required to respond quickly in order to obtain monetary rewards; by comparison, our task involved a forced-choice categorization with no explicit instruction for subjects to respond quickly. Nevertheless, it might have been expected that the RT results from Guitart-Masip et al. (2015) might generalize to a probabilistic categorization task such as the current paradigm. In our Experiment 2, (most) subjects got frequent reward during the reward-based trial blocks, and got no reward (or at best lack-of-punishment) during the punishment-based trial blocks, so arguably relative rate of reward changed across the course of the experiment. However, our RT analysis did not find significant effects of condition on RT nor any block-condition interactions. One possible explanation for this discrepancy is simply that our small sample size was underpowered to examine RT data. A second explanation might be that subjects in the current study viewed the no-feedback outcome as reinforcing, which meant that (for most subjects) relative rates of reward were similar across reward and punishment blocks, particularly since performance levels were approximately equal (Figures 2A, 8A). However, this explanation is not supported by the computational modeling, which suggested that, although the no-feedback outcome was positively-valued during punishment-based trials in the Punish-First condition, it was not positively-valued during punishment-based trials in the Reward-First condition. Future studies could be designed to further elucidate this issue, by explicitly varying the rate of reward in this task, perhaps especially following overtraining and the achievement of steady-state response behavior (Niv et al., 2007).

In summary, our study shows that probabilistic category learning is impacted by ordering of trials, and specifically by whether reward-based and punishment-based trials occur first or are intermixed. Our computational modeling suggests that these differences are reflected in the relative weighting of neutral feedback, and further suggests that early training on one type of trials, specifically reward-based trials, can create a difference in how neutral feedback is processed, relative to those receiving only punishment trials or intermixed reward-based and punishment-based trials. This may create conditions that facilitate subsequent learning of avoidance responses, when punishment-based learning is introduced, which in turn may suggest a way in which early experiences could confer later vulnerability to facilitated avoidance, which is a feature of anxiety disorders.

## Conflict of Interest Statement

The authors declare that the research was conducted in the absence of any commercial or financial relationships that could be construed as a potential conflict of interest.

## Footnotes

^1^We thank an anonymous reviewer of this article for the suggestion.
